# The diagnostic significance of cerebrospinal fluid cytology and circulating tumor DNA in meningeal carcinomatosis

**DOI:** 10.3389/fneur.2023.1076310

**Published:** 2023-03-03

**Authors:** Wei-Ying Di, Ya-Nan Chen, Yun Cai, Qiang Geng, Yan-Li Tan, Chun-Hui Li, Ya-Nan Wang, Yan-Hong Shang, Chuan Fang, Shu-Jie Cheng

**Affiliations:** ^1^Clinical Medical College, Hebei University, Baoding, China; ^2^Department of Neurology, Affiliated Hospital of Hebei University, Baoding, China; ^3^Department of Pathology, Affiliated Hospital of Hebei University, Baoding, China; ^4^Department of Neurosurgery, Affiliated Hospital of Hebei University, Baoding, China; ^5^Department of Oncology, Affiliated Hospital of Hebei University, Baoding, China; ^6^Hebei Key Laboratory of Precise Diagnosis and Treatment of Glioma, Baoding, China; ^7^Department of Hepatobiliary Surgery, Affiliated Hospital of Hebei University, Baoding, China

**Keywords:** meningeal carcinomatosis, lung adenocarcinoma, cerebrospinal fluid cytology examination, CtDNA, NGS

## Abstract

**Objective:**

The objective of this research is to investigate the clinical application value of cerebrospinal fluid (CSF) cytology and circulating tumor DNA (ctDNA) in lung adenocarcinoma (LUAD) meningeal metastasis-meningeal carcinomatosis (MC), and to further explore the possible molecular mechanisms and drug treatment targets of LUAD meningeal metastasis by next-generation sequencing (NGS).

**Methods:**

We retrospectively analyzed LUAD with MC in 52 patients. CSF cytology was carried out using the slide centrifugation precipitation method and May-Grüwald-Giemsa (MGG) staining. Tumor tissue, plasma and CSF ctDNA of some MC patients were detected by NGS.

**Results:**

Of the 52 MC patients, 46 (88.46%) were positive for CSF cytology and 34 (65.38%) were positive for imaging, with statistically significant differences in diagnostic positivity (*P* < 0.05). In 32 of these patients, CSF cytology, cerebrospinal fluid ctDNA, plasma ctDNA and MRI examination were performed simultaneously, and the positive rates were 84.38, 100, 56.25, and 62.50% respectively, the difference was statistically significant (*P* < 0.001). Analysis of the NGS profiles of tumor tissues, plasma and CSF of 12 MC patients: the mutated gene with the highest detection rate was epidermal growth factor receptor (EGFR) and the detection rate were 100, 58.33, and 100% respectively in tumor tissues, plasma and CSF, and there were 6 cases of concordance between plasma and tissue EGFR mutation sites, with a concordance rate of 50.00%, and 12 cases of concordance between CSF and tissue EGFR mutation sites, with a concordance rate of 100%. In addition, mutations not found in tissue or plasma were detected in CSF: FH mutation, SETD2 mutation, WT1 mutation, CDKN2A mutation, CDKN2B mutation, and multiple copy number variants (CNV), with the most detected being CDKN2A mutation and MET amplification.

**Conclusion:**

CSF cytology is more sensitive than traditional imaging in the diagnosis of meningeal carcinomatosis and has significant advantages in the early screening and diagnosis of MC patients. CSF ctDNA can be used as a complementary diagnostic method to negative results of CSF cytology and MRI, and CSF ctDNA can be used as an important method for liquid biopsy of patients with MC, which has important clinical significance in revealing the possible molecular mechanisms and drug treatment targets of meningeal metastasis of LUAD.

## 1. Introduction

Meningeal carcinomatosis (MC), also known as neoplastic meningitis (NM), refers to conditions in which a malignant tumor diffusely spreads to the leptomeninges, cerebrospinal fluid (CSF), and the subarachnoid space (1). MC is one of the most life-threatening types of brain metastasis with high morbidity and mortality as well as poor prognosis (2). Early diagnosis and prompt treatments are of great importance for improving the quality of life of MC patients. Currently, the diagnosis of MC is mainly based on observation of clinical symptoms and neuroimaging examinations. Given the complex, non-specific clinical manifestations and low imaging diagnostic sensitivity, MC can be misdiagnosed or missed, which delays early treatment. Detecting neoplastic cells in the CSF is the gold standard for diagnosing MC (3). Although repeated testing might improve the sensitivity of CSF cytology, ~25–30% of MC patients cannot be diagnosed by this method (4). Studies have shown that better diagnosis of MC can be achieved by CSF circulating tumor DNA (CSF ctDNA) detection, which more accurately recapitulates the genomic landscape of MC and might provide more insights regarding the molecular mechanism of tumorigenesis (5, 6).

Lung cancer is the cancer with the highest incidence and mortality in the world at present (7), and the 5-year survival rate of which is < 20% (8). Lung cancer is the most common malignant tumor causing MC, and 3–5% of non-small cell lung cancer patients will develop MC (9, 10). Lung adenocarcinoma (LUAD) is a common histological subtype of non-small cell lung cancer, accounting for about 40% of lung malignant tumors (11). Some MC patients were initially diagnosed in the Department of Neurology with initial symptoms in the nerve system. In this study, 12 patients were first diagnosed with headache, dizziness and/or nausea and vomiting in the Department of Neurology. Lumbar puncture was performed and increased intracranial pressure was detected. Abnormal cells were found in CSF cytology at the first examination. This study retrospectively analyzed the clinical, CSF cytological and imaging characteristics of MC patients with lung adenocarcinoma (LUAD) in our hospital. We compared the positive diagnosis rate of CSF cytology, traditional imaging-based method, plasma and CSF ctDNA detection, as well as integrated the next-generation sequencing (NGS) data from patient-derived tumor tissue, plasma and CSF. To further explore the early diagnosis methods and the possible pathogenesis of molecular mechanism of MC, and to provide a certain theoretical basis for drug treatment targets.

## 2. Subjects and methods

### 2.1. Subjects objects

Written informed consent was obtained from all patients or their legal guardians. The study protocol was approved by the Ethics Committee of the Affiliated Hospital of Hebei University, Hebei, China (protocol code HDFY-LL-2022-051). The MC patients with LUAD were enrolled from the affiliated Hospital of Hebei University from July 2019 to January 2022. The general data including sex, age, clinical manifestations, neuroimaging, CSF cytology, CSF examination were analyzed to examine the genetic characteristics of tumor tissue, CSF and plasma. Inclusion criteria were as follows (12, 13): (1) One or more of the following nervous system symptoms and physical signs: headache, nausea, vomiting, any kind of seizure, disturbance of consciousness, cranial nerve involvement (diplopia, hearing loss, vision loss, facial nerve palsy, etc), spinal symptoms (paraplegia, defecation disorders), etc. (2) LUAD was confirmed by histopathology. (3) Typical positive imaging morphological signs on magnetic resonance imaging (MRI): meningeal thickening or pia linear or cord-like enhancement. (4) Positive cytological examination results of CSF: detection of neoplastic cells features such as different size with irregular-shape, big nucleus with malignant signs such as lobulated state and malformed buds, increasing chromatin with basophilic coarse particles, mitotic activity with aberrant mitosis. (1, 2) items and (3) or (4) items were required for enrollment.

### 2.2. Method

#### 2.2.1. Examination method of CSF cytology

The Thermo Shandon Cytospin 4 cell centrifugal smear machine (Thermo Fisher Scientific, U. K.) was used to collect the cells by slide centrifugation precipitation method. 0.5 ml of fresh CSF was centrifuged at 650 RPM for 5 min. The CSF cells were centrifuged and precipitated on the slide and dried at room temperature. After May-Grüwald-Giemsa (MGG) staining, the morphology of the cells was observed under the light microscope and the cells were classified and counted.

#### 2.2.2. CSF ctDNA, plasma ctDNA and tissue gene detection by NGS

##### 2.2.2.1. DNA extraction and library construction

The collected samples and the corresponding negative control samples were centrifuged at room temperature, and the treated specimens were stored in pre-labeled freezing tubes in a −80°C refrigerator. The DNA was extracted with a MagMaxTM cell-free DNA isolation kit (Thermo Fisher) from the collected samples and was randomly sheared into 150–220 bp fragments using the Covaris system (Covaris). A total of 100 ng to 1.5 μg of sheared DNA prepared for amplification was purified with Agencourt AMPure XP SPRI beads (Beckman Coulter). The DNA was blunted with 5′-phosphorylated ends using the NEB Quick Blunting Kit and ligated to truncated PE P7 adaptors and barcoded P5 adaptors using NEBNext Quick Ligation Module (New England Biolabs). After cleanup with Agencourt AMPure XP SPRI beads and nick fill-in with Bst polymerase Large Fragment (New England Biolabs), the DNA fragments with adaptors were amplified and purified with the library amplification kit (KAPA Library Amplification Kit) according to the manufacturer's protocol.

##### 2.2.2.2. Probe enrichment

 DNA capture probe hybridization with library, add closed primers, hybridization solution and capture probe to genomic library to hybridize overnight; Washing and recovery of capture library products, use Wash Buffer to wash the hybridization products after capture to reduce the background of non-specific hybridization; Binding of capture library with streptavidin magnetic beads, mix the activated streptavidin magnetic beads with hybridization products Mix the activated streptavidin beads with the hybridization product and incubate overnight; Wash the bead capture library to remove the non-specific binding library.

##### 2.2.2.3. On-board sequencing

Prepare sequencing reagents according to the Hiseq 4000 User Guide, and put the flow cell with cluster on the machine (Hiseq 4000, Illumina). The paired-end program was selected for double-end sequencing, using the 520 panel as a tumor-associated gene capture probe. The sequencing process is controlled by Illumina's data collection software, and real-time data analysis is performed.

##### 2.2.2.4. Sequencing data analysis

**Figure 1:**
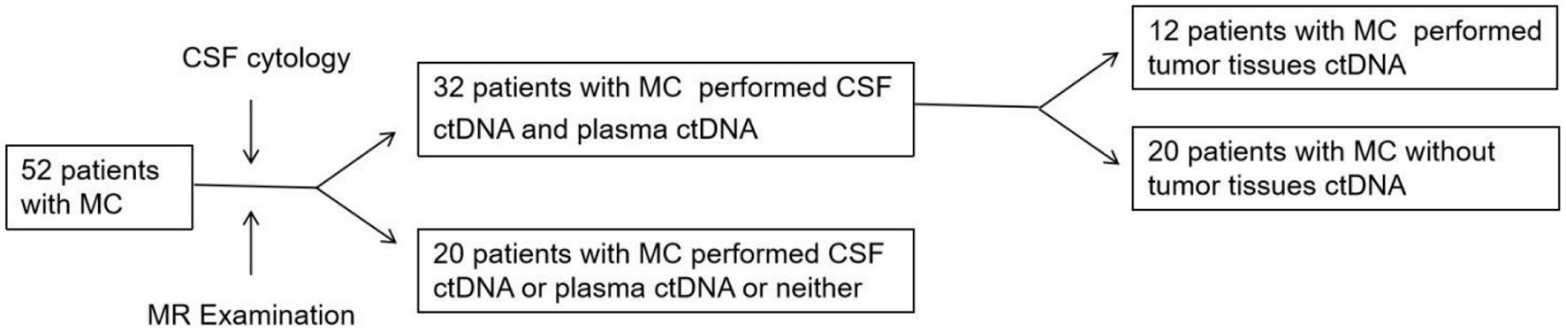
Study schema.

Using the human genome data hg19 as a reference analysis to compare the detected data, further screening and analysis of single nucleotide variants and copy number variant types, filtering mismatches and low quality sequences. The tumor-specific mutations were screened based on further comparison results from the thousand genomes database, dbSNP database, etc. The study schema as shown in Figure 1.

### 2.3. Statistical analysis

SPSS 25 (Statistical Package for the Social Sciences, version 25.0, IBM, New York, USA) was applied to the statistical analysis of the data: the observed values of the measurement data were tested for normality and chi-square test, and those with normal distribution were expressed as (mean ± standard deviation), those with non-normal distribution were expressed as (median, interquartile spacing), and the count data were expressed as rate (%), the chi-square test was used to compare the differences between groups of count data, and the *Q*-test was used to compare multiple rates of the collocation design, and *P* < 0.05 was considered a statistically significant difference.

## 3. Results

### 3.1. General data

General data of 52 patients with MC (Table 1).

**Table 1:** General data of 52 patients with MC.

		**The number of cases (*n*)**	**Percentage (%)**
Sex	Male	22	42.30
	Female	30	57.70
Clinical manifestation	Headache, nausea and vomiting	45	86.54
	Dizziness	19	36.54
	Cognitive impairment	9	17.31
	Conscious disturbance	6	11.54
	Blurred vision	5	9.62
	Psychological and behavioral abnormalities	2	3.85
	Fever	2	3.85
	Epileptic seizure	2	3.85
	Abducent paralysis	2	3.85
	Numbness and paralysis of limbs	1	1.92
	Neck stiffness	40	76.92
Head MRI	Meningeal enhancement	12	23.08
	Brain parenchyma metastasis	8	15.38
	Meningeal enhancement with brain parenchyma metastasis	22	42.31
	No abnormal change	10	19.23
Cerebrospinal fluid	**Intracranial pressure**		
	•Normal (80~180 mmH_2_O)	7	13.46
	•Increased (>180 mmH_2_O)	45	86.54
	**White blood cell count**		
	•0~5 × 10^6^/L	17	32.69
	•>5 × 10^6^/L	35	67.31
	**Protein**		
	• < 0.44 g/L	23	44.23
	•0.44~1.3 g/L	29	55.77
	**Glucose**		
	•1.4~2.3 mmol/L	35	67.31
	•>2.3 mmol/L	17	32.69
	**Cytology**		
	•Activated monocytosis	38	73.08
	•Lymphocyte response mainly	34	65.38
	•Neutrophil response mainly	1	1.92
	•Monocyte response mainly	17	32.69

### 3.2. Sensitivity comparison of CSF cytology and MRI positive rate

Twenty eight cases were positive for both examinations, 18 cases were tested positive for CSF cytology and negative for MRI, while six cases were tested positive for MRI but negative in CSF cytology analysis. The positive rate of CSF cytology was 88.46% (46/52), which is significantly higher than MRI positive rate (65.38%, 34/52, *P* < 0.05) (Table 2).

**Table 2:** Comparison of positive rate of CSF cytology and head MRI of 52 patients with MC.

**Detection method**	**Number of positive cases (*n*)**	**Total number of cases (*n*)**	**Positive rate (%)**
CSF cytology	46	52	88.46
Head MRI	34	52	65.38
χ^2^			5.04
*P*			0.023

### 3.3. Sensitivity comparison of CSF cytology, CSF ctDNA, plasma ctDNA and head MRI

Thirty two patients underwent CSF cytology, CSF ctDNA, plasma ctDNA and head MRI simultaneously, 27 were tested positive for CSF cytology (84.38%, 27/32), all 32 patients were tested positive for CSF ctDNA (100%; 32/32), 18 were tested positive for plasma ctDNA (56.25%, 18/32), 20 were tested positive for MRI (62.50%, 20/32). The positive rates of all four methods were statistically significant (*P* < 0.001) (Table 3).

**Table 3:** Comparison of positive rate of CSF cytology, CSF ctDNA, plasma ctDNA and head MRI of 32 patients with MC.

**Detection method**	**Number of positive cases (*n*)**	**Total number of cases (*n*)**	**Positive rate (%)**
CSF cytology	27	32	84.38
CSF ctDNA	32	32	100
Plasma ctDNA	18	32	56.25
Head MRI	20	32	62.50
*Q*			30.55
*P*			0.000

### 3.4. Next generation sequencing

Next generation sequencing of tumor tissue, plasma and CSF were carried out in total of 12 patients. The most frequently mutated gene was found to be EGFR, with a positive diagnostic rate of 100% in both CSF and tumor tissue, and a rate of 58.33% in patient plasma. The same EGFR mutation variant was found in paired plasma and tumor tissue samples of 6 patients (6/12); analysis of the corresponding mutations in the CSF ctDNA NGS data revealed good concordance with tumor samples, the same EGFR site mutation was identified in all twelve paired of CSF and tumor tissue samples. Moreover, many unique mutations were only found in CSF samples, but not in the tumor tissue and plasma samples, which includes FH mutation, SETD2 mutation, WT1 mutation, CDKN2A mutation, CDKN2B mutation. CNV was also seen. The most frequently found mutation were CDKN2A mutation and MET amplification (Table 4).

**Table 4:** Results of pathological tissue ctDNA, plasma ctDNA and CSF ctDNA of 12 patients with MC.

**No**.	**Pathological tissue ctDNA**	**Plasma ctDNA**	**CSF ctDNA**
N01	EGFR 19del, PIK3CA	Negative	EGFR 19del
N02	EGFR L858R	EGFR L858R	EGFR L858R, SETD2
N03	EGFR L858R, TP53	EGFR L858R, TP53	EGFR L858R, TP53
N04	EGFR L858R	RB1	EGFR L858R, CDKN2A CDKN2B
N05	EGFR L858R	EGFR L858R, TP53	EGFR L858R, TP53
N06	EGFR L858R, TP53	EGFR L858R, TP53	EGFR L858R, TP53
N07	EGFR L858R	Negative	EGFR L858R, CDKN2A, FH, TP53, WT1
N08	EGFR 19del	Negative	EGFR 19del, CDKN2A CDKN2B TP53
N09	EGFR L858R	EGFRT790M	EGFR L858R
N10	EGFR L858R	EGFR L858R, PIK3CA	EGFR L858R
N11	EGFR L858R	Negative	EGFR L858R, TP53
N12	EGFR 19del, TP53	EGFR 19del	EGFR 19del, TP53

## 4. Discussion

Malignant tumors can be life-threatening. Because of the continuous improvement of diagnostic techniques and the prolongation of the survival time in cancer patients, the incidence of MC increased year by year. MC can occur in almost all malignant tumors. The cancer types with highest MC incidence rate are melanoma (23%) lung cancer (9–25%) and breast cancer (5%) (14). A total of 52 patients with MC were analyzed in this study, all of which derived from LUAD.

Meningeal cancer is more common in middle-aged and more elderly people, with either acute or subacute onset (15). Most patients have clinical manifestations like cranial hypertension symptoms, headache, nausea and vomiting. With the progress of the disease, the cerebral hemispheres and cranial nerves are gradually involved, which leads to cognitive disorders, mental abnormalities, personality changes, seizures, diplopia, extraocular muscle paralysis and other symptoms (14, 16). Most of the 52 patients involved in this study was middle-aged to elderly people and had subacute onset, mainly presented with cranial hypertension, cranial nerve involvement, and cognitive impairment, which were consistent with literature reports (15). However, many MC patients also presented with atypical clinical manifestations, such as meningitis, cerebrovascular disease, sinus thrombosis, or peripheral nerve injury. In this study, there were two cases with meningitis, one case each with cerebral infarction, venous sinus thrombosis and peripheral nerve injury.

The typical features of meningeal enhancement in MC are as follows: diffuse or multiple thickening of pia with enhancement; or local nodular enhancement of the pia mater. The imaging characteristics of the patients in this study were typical, and the meninges appeared to be normal by plain scan, suggesting that the sensitivity of MRI scan in the diagnosis of MC was low, which may be related to the fact that cancer cells mainly invaded along the meninges and distributed diffusely, and there was no obvious contrast between the diseased tissues and the adjacent CSF. Contrast-enhanced cranial MRI is the first choice for evaluating patients with suspected cancerous meningitis, but some studies have shown that the incidence of false negative results is still as high as 30% (17). The sensitivity of cranial enhanced MRI in this study was 65.38%, which is consistent with the results of previous studies (18). In this study, 67.31% of the cases had increased white blood cell count in cerebrospinal fluid, 55.77% had increased protein, and 67.31% had decreased glucose, suggesting an inflammatory environment in the meninges. And 65.38% of the cases were characterized by mainly lymphocyte response, while 73.08% showed activated monocytes Increased, supporting that increased activated mononuclear cell number is one of the CSF characteristics of central nervous system cancers.

**Figure 2:**
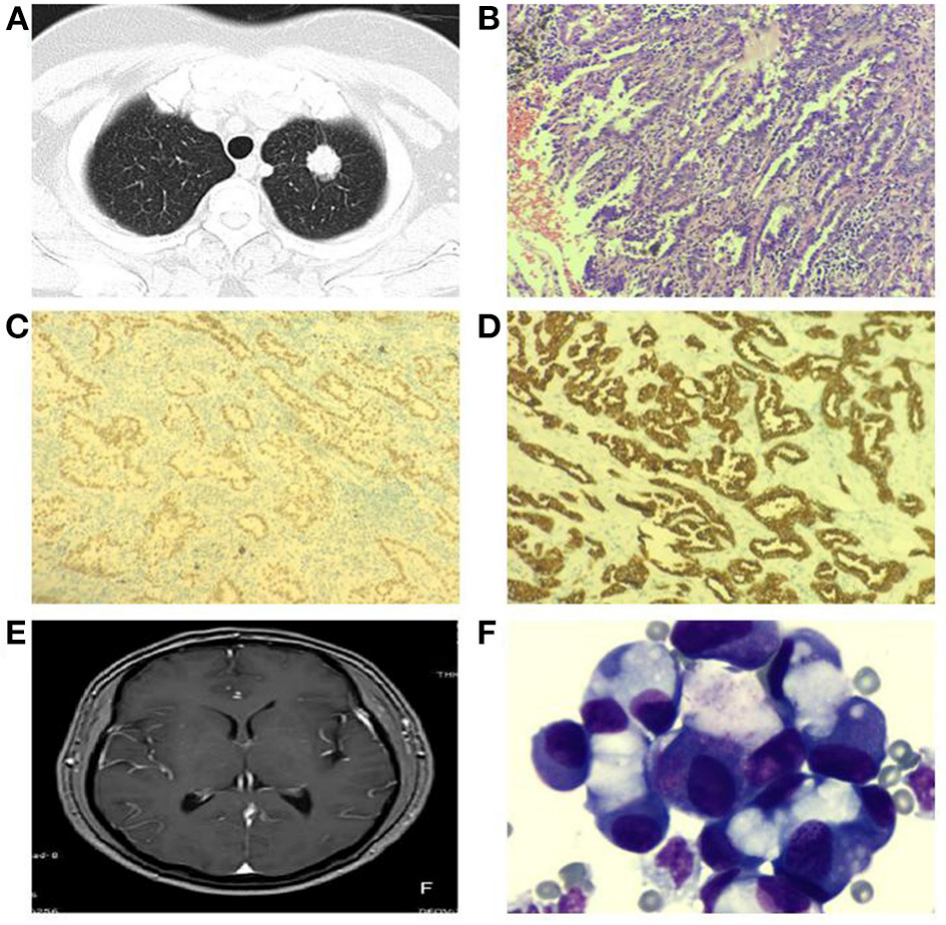
Typical case shows: patient, male, 66 years old, left lung mass **(A)** visible at chest CT, lung cancer tissue HE staining **(B)**, positive expression of cytokeratin 7 (CK7) in cell membrane of lung cancer tissue **(C)**, positive expression of thyroid transcription factor-1 (TTF-1) in cell nucleus of lung cancer tissue **(D)**; 1 year later, the patient developed headache, nausea and vomiting, no pivot meningeal enhancement was seen in the head MRI enhancement **(E)**, CSF cytology showed a large number of tumor cells **(F)** [**(B–D)**:200 × ; **(F)**:1000 × ].

Due to the lack of specificity of MC clinical manifestations and low sensitivity of neuroimaging, it is more likely to cause misdiagnosis and missed diagnosis, which brings difficulties to the early diagnosis and treatment of patients with MC (5). In 2021, the Consensus of Chinese MC Experts recommended CSF cytology as the first choice for MC diagnosis, considering that it is a simple and low-cost examination method, with higher sensitivity and specificity than neuroimaging and clinical symptom-based methods (19). ctDNA has emerged as a promising blood-based biomarker for monitoring disease status of patients with advanced cancers. One group reported that the sensitivity of CSF cytology of the first lumbar puncture is 50–60%, but repetitive collection can increase the sensitivity to 80% (3). The results suggested that 46 cases were positive, with a sensitivity of 88.46%. Tumor cells were found for the first time in all 46 patients, which was higher than earlier research from the literature, which may be associated with the short time from sample collection to examination and the detection method (20). The results of this study showed that CSF cytology was more sensitive than traditional imaging-based diagnosis methods, which was consistent with previous studies (5) (The introduction and related examination results of a typical case are attached in this article, as shown in Figure 2).

However, CSF cytology has its own limitations and might results in false negative results. Liquid biopsy method overcomes many limitations of the traditional tissue biopsy, such as tumor heterogeneity, invasive operation risk, sample preparation error, etc., and therefore has great potential in tumor diagnosis and screening (21–23). CtDNA is the circulating DNA released into body fluids from cancer cells undergoing apoptosis or necrosis, in which the genetic alterations could be detected by pcr-based or NGS based methods (24, 25). Plasma tumor-derived ctDNA has been used to detect a variety of different types of tumors, however, plasma ctDNA detection is not very effective in patients with brain metastases (6, 26, 27). Cancer cells that spread to the meninges are in direct contact with the CSF, and the ctDNA fragments released can enter the CSF directly. However, due to the existence of the blood-brain barrier, it is hard for the ctDNA in the CSF to enter the blood (28). Therefore, CSF ctDNA has the potential to be utilized as an important marker for MC diagnosis. In this study, all four diagnostic methods (CSF cytology, CSF ctDNA, plasma ctDNA and head MRI) were carried out in 32 patients, and with the highest positive rate in CSF ctDNA analysis (100%). The difference was statistically significant (*P* < 0.05), suggesting that the sensitivity of CSF was higher than that of peripheral blood, which was consistent with previous findings (7). CSF cytology is more sensitive than traditional imaging approaches for MC diagnosis. CSF ctDNA analysis can be considered as a supplementary method to confirm negative results from CSF cytology and MRI analysis.

CtDNA carries a plethora of tumor gene mutations and other information. Multiple studies have shown that ctDNA in peripheral blood can reflect the same gene mutational landscape in solid tumor tissues, and is an important monitoring index for evaluation of treatment effect, clinical follow-up and identification of drug resistance mechanisms (29). For patients with brain tumors or brain metastases, the level of ctDNA in CSF is higher than that in their peripheral blood (21, 30). CtDNA examination in CSF not only contributes to the diagnosis of MC, but also helps to further understand the gene mutation characteristics of intracranial metastases, as well as helps to uncover the mechanisms for meningeal metastasis and to identify novel therapeutic targets. Next generation sequencing carried out in tissue, plasma, CSF of a total of 12 patients. All 12 patients carried EGFR mutation. Analysis of the corresponding mutations in the ctDNA NGS data revealed good concordance with tumor sample, the same mutation variant of EGFR was found in all paired CSF and tumor tissue samples. Moreover, many unique mutations were only found in CSF tissue samples, including FH mutation, SETD2 mutation, WT1 mutation, CDKN2A mutation, CDKN2B mutation, and CNV, among which the most frequently found mutation were CDKN2A mutation and MET amplification.

Cell cycle-dependent kinase inhibitor gene (CDKN2A) is a tumor suppressor gene that encodes two cell cycle suppressor proteins, p16^INK4a^ and p14^ARF^, to regulate cell cycle (31). Homozygous loss of CDKN2A, promoter methylation or gene point mutation can lead to loss of p16 and p14 expression, which is closely related to tumor prognosis (32). Studies have reported that CDKN2A methylation is an independent indicator of poor prognosis in patients with non-small cell lung cancer (NSCLC) (33). Other studies have shown that lung cancer patients with EGFR mutation combined with CDKN2A deletion mutation have poor response to EGFR-TKI drugs (34). In this study, CDKN2A mutation was detected in the CSF of patient 4, 7, and 8, but not in the tissue or plasma in those patients. Those patients had unsatisfied response to EGFR tyrosine kinase inhibitor Osimertinib, which might be associated with the CDKN2A mutation detected in the CSF. CNV are one of the major genomic variations, which are reproducible from cell to cell. Ni et al. (35) found that the CNV combinations of certain gene loci (such as c-Myc, TERT, HLA) may lead to specific gene expression, resulting in selective advantage in metastasis. MET amplification was the most frequently detected CNV in our study. MET is a proto-oncogene commonly found in lung cancer patients. MET amplification is associated with poor prognosis of NSCLC patients. Okuda et al. (36) analyzed pathological sections of 213 NSCLC patients and found that increased MET copy number was an important indicator for poor prognosis of NSCLC patients after surgery. Cappuzzo et al. (37) showed that the prognosis of NSCLC patients with MET amplification may be related to their average gene copy number. When MET binds to its ligand human hepatocyte growth factor (HGF) in NSCLC, it can activate a variety of intracellular signaling pathways, such as PI3K-AKT, RAS-MAPK, and STAT3 pathways (38, 39), affecting the proliferation, survival, apoptosis, invasion, migration and angiogenesis of NSCLC cells (40). Both CDKN2A abnormalities and MET amplification are associated with the disease progression and poor prognosis of lung cancer patients. However, it remains unclear whether CDKN2A abnormalities and MET amplification are involved in the process of brain metastasis of LUAD and how it affects the prognosis of meningeal cancer patients. Further studies on specific gene mutations and CNVs in patients with lung cancer brain metastases might provide more insights on the molecular mechanism of lung cancer brain metastases.

In conclusion, the value of ctDNA detection in CSF of patients with meningeal carcinoma provides more clinical benefits than that in patient plasma, which more comprehensively reflect the mutational burden of intracranial tumor tissue. If implemented well, performed at high quality, it can be a reliable, robust and accurate test which is of great significance for revealing the molecular mechanisms of meningeal metastasis.

## Data availability statement

The data presented in the study are deposited in the Baidu Netdisk, here's the link: https://pan.baidu.com/s/1sAOX62o339Y0w3ERYuYWXg?pwd=stf9, accession number: stf9.

## Ethics statement

The studies involving human participants were reviewed and approved by Affiliated Hospital of Hebei University. The patients/participants provided their written informed consent to participate in this study. Written informed consent was obtained from the individual(s) for the publication of any potentially identifiable images or data included in this article.

## Author contributions

W-YD, Y-NC, YC, CF, and S-JC study conception, design, analysis and interpretation of data, and critical revision of the manuscript for important intellectual content. W-YD, Y-NC, YC, and QG acquisition of data and drafting of the manuscript. Y-NC, YC, QG, Y-LT, C-HL, Y-NW, and Y-HS statistical analysis. All authors contributed to the article and approved the submitted version.

## References

[1] Jorda M Ganjei-Azar P Nadji M. Cytologic characteristics of meningeal carcinomatosis: increased diagnostic accuracy using carcinoembryonic antigen and epithelial membrane antigen immunocytochemistry. Arch Neurol. (1998) 55:181–4. 9482359 10.1001/archneur.55.2.181

[2] Chamberlain MC. Neoplastic meningitis. Curr Neurol Neurosci Rep. (2008) 8:249–58. 10.1007/s11910-008-0038-6 18541120

[3] Nayar G Ejikeme T Chongsathidkiet P Elsamadicy AA Blackwell KL Clarke JM. Meningeal disease: current diagnostic and therapeutic strategies. Oncotarget. (2017) 8:73312–28. 10.18632/oncotarget.20272 29069871 PMC5641214

[4] Azevedo CR Cruz MRS Chinen LTD Peres SV Peterlevitz MA Pereira AE. Meningeal carcinomatosis in breast cancer: prognostic factors and outcome. J Neuro-Oncol. (2011) 104:565–72. 10.1007/s11060-010-0524-y 21234642

[5] Zhao Y He JY Zou YL Guo XS Cui JZ Guo L. Evaluating the cerebrospinal fluid ctDNA detection by next-generation sequencing in the diagnosis of meningeal Carcinomatosis. BMC Neurol. (2019) 19:331. 10.1186/s12883-019-1554-5 31856745 PMC6924020

[6] Li YS Jiang BY Yang JJ Zhang XC Zhang Z Ye JY. Unique genetic profiles from cerebrospinal fluid cell-free DNA in meningeal metastases of EGFR-mutant non-small-cell lung cancer: a new medium of liquid biopsy. Ann Oncol. (2018) 29:945–52. 10.1093/annonc/mdy009 29346604

[7] Bray F Ferlay J Soerjomataram I Siegel RL Torre LA Jemal A. Global cancer statistics 2018: GLOBOCAN estimates of incidence and mortality worldwide for 36 cancers in 185 countries. CA Cancer J Clin. (2018) 68:394–424. 10.3322/caac.21492 30207593

[8] Herbst RS Morgensztern D Boshoff C. The biology and management of non-small cell lung cancer. Nature. (2018) 553:446–54. 10.1038/nature25183 29364287

[9] Shi Y Sun Y Yu J Ding C Ma Z Wang Z. China experts consensus on the diagnosis and treatment of brain metastases of lung cancer (2017 version). Zhongguo Fei Ai Za Zhi. (2017) 20:1–13. 10.1111/ajco.12608 28103967 PMC5973287

[10] Jiang BY Li YS Guo WB Zhang XC Chen ZH Su J. Detection of driver and resistance mutations in leptomeningeal metastases of NSCLC by next-generation sequencing of cerebrospinal fluid circulating tumor cells. Clin Cancer Res. (2017) 23:5480–8. 10.1158/1078-0432.CCR-17-0047 28606923

[11] Chen Z Fillmore CM Hammerman PS Kim CF Wong K-K. Non-small-cell lung cancers: a heterogeneous set of diseases. Nat Rev Cancer. (2014) 14:535–46. 10.1038/nrc3775 25056707 PMC5712844

[12] Qian M Ren H Qu T Lu Z Zou Y He J. Spectrum of clinical, neuroimaging, and cerebrospinal fluid features of adult neurocutaneous melanocytosis. Eur Neurol. (2018) 80:1–6. 10.1159/000488687 30007971

[13] Ren H Zou Y Zhao Y Li J Han X He J. Cerebrospinal fluid cytological diagnosis in multiple myeloma with meningeal involvement: a report of two cases. Diagn Cytopathol. (2017) 45:66–8. 10.1002/dc.23600 27628930

[14] Thakkar JP Kumthekar P Dixit KS Stupp R Lukas RV. Meningeal metastasis from solid tumors. J Neurol Sci. (2020) 411:116706. 10.1016/j.jns.2020.116706 32007755

[15] Zhang QQ He JY Liu X Grant R Naylor B Greenberg HS. Clinical analysis of 94 patients with meningeal carcinomatosis. Chin J Neuropsych Disord. (2015) 41:715–9. 10.3969/j.issn.1002-0152.2015.12.003

[16] Chowdhary S Damlo S Chamberlain MC. Cerebrospinal fluid dissemination and neoplastic meningitis in primary brain tumors. Cancer Control J Moffitt Cancer Center. (2017) 24:S1–S16. 10.1177/107327481702400118 28557973

[17] Fracp RJFM George Krol MD Deangelis DLM. Neuroimaging and cerebrospinal fluid cytology in the diagnosis of meningeal metastasis. Ann Neurol. (1995) 38:51–7. 7611725 10.1002/ana.410380111

[18] Singh SK Agris JM Leeds NE Ginsberg LE. Intracranial leptomeningeal metastases: comparison of depiction at FLAIR and contrast-enhanced MR imaging. Radiology. (2000) 217:50–3. 10.1148/radiology.217.1.r00oc3550 11012422

[19] Chamberlain M Junck L Brandsma D Soffietti R Rudà R Raizer J. Meningeal metastases: a RANO proposal for response criteria. Neuro Oncol. (2017) 19:484–92. 10.1093/neuonc/now183 28039364 PMC5464328

[20] Su X. Appeal for clinical cerebrospinal fluid cytological examination with slide centrifugal precipitation method. J Clin Neurol. (2009) 04:312.

[21] De Mattos-Arruda L Mayor R Ng CKY Weigelt B Martínez-Ricarte F Torrejon D. Cerebrospinal fluid-derived circulating tumour DNA better represents the genomic alterations of brain tumours than plasma. Nat Commun. (2015) 6:8839. 10.1038/ncomms9839 26554728 PMC5426516

[22] Lim M Kim CJ Sunkara V Kim MH Cho YK. Liquid biopsy in lung cancer: Clinical applications of circulating biomarkers (CTCs and ctDNA). Micromachines. (2018) 9:100. 10.3390/mi9030100 30424034 PMC6187707

[23] Jamal-Hanjani M Wilson GA Horswell S Mitter R Sakarya O Constantin T. Detection of ubiquitous and heterogeneous mutations in cell-free DNA from patients with earlystage non-small-cell lung cancer. Ann Oncol. (2016) 27:862–7. 10.1093/annonc/mdw037 26823523

[24] Siravegna G Mussolin B Venesio T Marsoni S Seoane J Dive C. How liquid biopsies can change clinical practice in oncology. Ann Oncol. (2019) 30:1580–90. 10.1093/annonc/mdz227 31373349

[25] Heitzer E Haque IS Roberts CES Speicher MR. Current and future perspectives of liquid biopsies in genomics-driven oncology. Nat Rev Genet. (2019) 20:71–88. 10.1038/s41576-018-0071-5 30410101

[26] Sindeeva OA Verkhovskii RA Sarimollaoglu M Afanaseva GA Fedonnikov AS Osintsev EY. New frontiers in diagnosis and therapy of circulating tumor markers in cerebrospinal fluid *in vitro* and *in vivo*. Cells. (2019) 8:1195. 10.3390/cells8101195 31581745 PMC6830088

[27] Zhao J Ye X Xu Y Chen M Zhong W Sun Y. EGFR mutation status of paired cerebrospinal fluid and plasma samples in EGFR mutant non-small cell lung cancer with meningeal metastases. Cancer Chemother Pharmacol. (2016) 78:1305–10. 10.1007/s00280-016-3155-y 27770237

[28] Bettegowda C Sausen MJ Leary R Kinde I Wang Y Agrawal N. Detection of circulating tumor DNA in early-and late-stage human malignancies. Sci Transl Med. (2014) 6:224–47. 10.1126/scitranslmed.3007094 24553385 PMC4017867

[29] Liao BC Lee JH Lin CC Chen YF Chang CH Ho CC. Epidermal growth factor receptor tyrosine kinase inhibitors for non-small-cell lung cancer patients with meningeal carcinomatosis. J Thorac Oncol. (2015) 10:1754–61. 10.1097/JTO.0000000000000669 26334749

[30] Li M Dressman D He Y Li M Shen D Szabo S. Detection and quantification of mutations in the plasma of patients with colorectal tumors. Proc Natl Acad Sci U S A. (2005) 102:16368–73. 10.1073/pnas.0507904102 16258065 PMC1283450

[31] Lutful Kabir FM Agarwal P Deinnocentes P Zaman J Bird AC Bird RC. Novel frameshift mutation in the p16/INK4A tumor suppressor gene in canine breast cancer alters expression from the p16/INK4A/p14ARF locus. J Cell Biochem. (2013) 114:56–66. 10.1002/jcb.24300 22833492

[32] Aoude LG Wadt KA Pritchard AL Hayward NK. Genetics of familial melanoma: 20 years after CDKN2A. Pigment Cell Melanoma Res. (2015) 28:148–60. 10.1111/pcmr.12333 25431349

[33] Lou-Qian Z Rong Y Ming L Xin Y Feng J Lin X. The prognostic value of epigenetic silencing of p16 gene in NSCLC patients: a systematic review and meta-analysis. PLoS ONE. (2013) 8:e54970. 10.1371/journal.pone.0054970 23372805 PMC3555860

[34] Jiang J Gu Y Liu J Wu R Fu L Zhao J. Coexistence of p16/CDKN2A homozygous deletions and activating EGFR mutations in lung adenocarcinoma patients signifies a poor response to EGFR-TKIs. Lung Cancer. (2016) 102:101–7. 10.1016/j.lungcan.2016.10.015 27987577

[35] Ni X Zhuo M Su Z Duan J Gao Y Wang Z. Reproducible copy number variation patterns among single circulating tumor cells of lung cancer patients. Proc Natl Acad Sci U S A. (2013) 110:21083–8. 10.1073/pnas.1320659110 24324171 PMC3876226

[36] Okuda K Sasaki H Yukiue H Yano M Fujii Y. Met gene copy number predicts the prognosis for completely resected non-small cell lung cancer. Cancer Sci. (2008) 99:2280–5. 10.1111/j.1349-7006.2008.00916.x 19037978 PMC11159911

[37] Cappuzzo F Marchetti A Skokan M Rossi E Gajapathy S Felicioni L. Increased MET gene copy number negatively affects survival of surgically resected non-small-cell lung cancer patients. J Clin Oncol. (2009) 27:1667–74. 10.1200/JCO.2008.19.1635 19255323 PMC3341799

[38] Goyal L Muzumdar MD Zhu AX. Targeting the HGF/c-MET pathway in hepatocellular carcinoma. Clin Cancer Res. (2013) 19:2310–8. 10.1158/1078-0432.CCR-12-2791 23388504 PMC4583193

[39] Trovato M Torre ML Ragonese M Simone A Scarfì R Barresi V. HGF/c-met system targeting PI3K/AKT and STAT3/phosphorylated-STAT3 pathways in pituitary adenomas: an immunohistochemical characterization in view of targeted therapies. Endocrine. (2013) 44:735–43. 10.1007/s12020-013-9950-x 23576023

[40] Favoni RE Alama A. Preclinical strategies targeted at non-small-cell lung cancer signaling pathways with striking translational fallout. Drug Discov Today. (2013) 18:11–24. 10.1016/j.drudis.2012.07.011 22885521

